# Patient and public involvement in medical performance processes: A systematic review

**DOI:** 10.1111/hex.12852

**Published:** 2018-12-11

**Authors:** Mirza Lalani, Rebecca Baines, Marie Bryce, Martin Marshall, Sol Mead, Stephen Barasi, Julian Archer, Samantha Regan de Bere

**Affiliations:** ^1^ Department of Primary Care and Population Health University College London London UK; ^2^ Collaboration for the Advancement of Medical Education Research and Assessment Faculty of Medicine and Dentistry University of Plymouth Plymouth UK; ^3^ General Medical Council, Registration and Revalidation Directorate London UK; ^4^ NHS England London and Southeast Regions, Regional Medical Directorate London UK; ^5^ General Medical Council, Registration and Revalidation Directorate (Wales) Wales UK

**Keywords:** medical performance, patient involvement

## Abstract

**Background:**

Patient and public involvement (PPI) continues to develop as a central policy agenda in health care. The patient voice is seen as relevant, informative and can drive service improvement. However, critical exploration of PPI's role within monitoring and informing medical performance processes remains limited.

**Objective:**

To explore and evaluate the contribution of PPI in medical performance processes to understand its extent, purpose and process.

**Search strategy:**

The electronic databases PubMed, PsycINFO and Google Scholar were systematically searched for studies published between 2004 and 2018.

**Inclusion criteria:**

Studies involving doctors and patients and all forms of patient input (eg, patient feedback) associated with medical performance were included.

**Data extraction and synthesis:**

Using an inductive approach to analysis and synthesis, a coding framework was developed which was structured around three key themes: issues that shape PPI in medical performance processes; mechanisms for PPI; and the potential impacts of PPI on medical performance processes.

**Main results:**

From 4772 studies, 48 articles (from 10 countries) met the inclusion criteria. Findings suggest that the extent of PPI in medical performance processes globally is highly variable and is primarily achieved through providing patient feedback or complaints. The emerging evidence suggests that PPI can encourage improvements in the quality of patient care, enable professional development and promote professionalism.

**Discussion and conclusions:**

Developing more innovative methods of PPI beyond patient feedback and complaints may help revolutionize the practice of PPI into a collaborative partnership, facilitating the development of proactive relationships between the medical profession, patients and the public.

## 1 BACKGROUND

Internationally, patient and public involvement (PPI) in health care has been described as “central to the reform of Western economies” and its growth reflects the realization that the patient voice is relevant, informative and drives service improvement.^1^, ^2^, ^3^ Whilst there is a developing academic literature base for PPI in health services, research and education, little is known of the evidence for PPI in the sphere of professional, and specifically medical, performance.

The last 20 years have witnessed a significant shift towards greater public accountability from health service organizations and health professionals, a possible consequence of which is the increased prominence of PPI. In the United Kingdom, the Health and Social Care Act (2001) introduced statutory PPI in service development, delivery and evaluation and is seen as a pivotal juncture in the evolution of PPI in health care–related research and education.^4^, ^5^ In the United States, the *Hospital Consumer Assessment of Health Providers and Systems* (HCAPHS) surveys were thought to have stimulated greater PPI in health care. However, Australia, New Zealand, Canada and most European countries (Norway and the Netherlands aside) have limited systems to capture and measure patient experience at a national level, although regional and local arrangements may exist.^6^, ^7^

However, in contrast with the developing evidence base for the impacts of PPI in health services, far less is known about the inclusion of PPI in medical performance processes and its impacts in this professional sphere. Globally, recertification, re‐licensure and revalidation are terms that have been used to describe a process by which a doctor's performance is continually assessed, ensuring they are up to date and fit to practice, reassuring patients and the public that they remain competent throughout their careers.^8^, ^9^ Internationally, PPI in medical performance processes varies considerably. Several countries have appointed members of the public to licensing boards and professional associations, a trend borne from a greater societal and governmental desire for accountability from the medical profession.^10^, ^11^ Additionally, despite countries adopting recertification or re‐licensure of doctors,^12^ the PPI element in these processes is seldom reported in the academic literature. For example, in Belgium, evidence for continuing medical education (CME) involves a review of complaints or compliments.^13^ The College of Physicians and Surgeons of Alberta, Canada, the statutory medical registration body for the province, has adopted a multi‐source feedback (MSF) system for all physicians/surgeons in its jurisdiction.^14^ In the USA, the American Board of Medical Specialties maintenance of certification (MOC) programme requires the submission of a patient survey as a sub‐component of demonstrating competence with interpersonal and communication skills.^15^

Though examples of PPI within medical performance processes and regulation are evident internationally, much of the evidence in this domain originates from studies of PPI in medical revalidation in the UK.^16^ In 2012, medical revalidation was mandated for all doctors in the UK. The Picker Institute's report, *The Patient Voice in Revalidation,* viewed revalidation as a necessary patient focussed reform, making patients its beneficiaries by representing them in some of its key tenets: “reassure the public,” “ensure patient safety” and “public trust.”^17^ Whilst improved patient care is seen as the purpose of revalidation, PPI in the infrastructure, systems and processes of revalidation is currently limited to patient feedback on an individual doctor and disparate lay representation on local and national steering, advisory and implementation groups.^18^ Individual doctors are required to submit and (reflect upon) patient feedback as part of their appraisal portfolio, once in their revalidation cycle (normally every 5 years).^19^ A recent report evaluating medical revalidation in the UK found that overall, PPI in revalidation was viewed favourably by most stakeholders but there remained some confusion over its intended purpose and models of delivery.^20^

Against this background, in this review we aimed to establish the contribution of PPI in medical performance processes internationally by exploring how PPI is operationalized, establishing the gateways and barriers to PPI in medical performance processes and understanding how PPI in all forms of patient input is influential in changing or modifying the practice of doctors.

## 2 METHODS

### 2.1 Design

The review followed the Preferred Reporting Items for Systematic Reviews and Meta‐Analysis (PRISMA) guidelines,^21^ and Popay's *Guidance on the conduct of narrative synthesis in systematic reviews*.^22^ The review protocol is published on the PROSPERO website (registration number CRD42016035969).^23^

### 2.2 Eligibility criteria

There is large conceptual variation around the terms used to describe PPI. The terms “patients” and “public” are often used interchangeably as are “involvement,” “participation” and “engagement.”^24^ This was considered when developing the search terms facilitated by the PICOS (population, intervention, comparator, outcome, study design) framework (Table 1).^25^ We assessed studies against eligibility criteria based on the PICOS elements. For the “population,” studies involving medical regulation stakeholders such as the public, patients and doctors, as well as the infrastructure for regulation, the national, regional (or federal) medical regulators or boards, professional bodies (eg, Royal colleges) and patient groups were included. In terms of the intervention, we included studies comprising all forms of patient input including lay representation, patient feedback, online reviews, information from patient surveys (experience/satisfaction), compliments and complaints. Through scoping, it was noted that no studies identified regulation as a specific outcome; hence, criteria were broadened to include outcomes relating to doctor/physician performance. Study design was not used as a basis for exclusion; however, we did exclude reviews, commentaries, opinion papers, etc., as well as studies associated with PPI in clinical decision making, research, education, health service provision or in the regulation/performance of other health professionals. Studies assessing the validity of patient feedback/satisfaction/experience tools were also excluded.

**Table 1:** Summary of review search strategy and eligibility criteria

Databases	1. MEDLINE 2. PsycINFO 3. Google Scholar	
Other Sources	Forward/ancestry citations from reviewed papers	
Key terms	*Population (P)*: *Intervention (I*): *Comparator (C):* *Outcome (O):*	“doctor” OR “physician” AND “patient involvement/engagement/participation/feedback/experience/satisfaction/survey/service user/lay/co‐production” OR “public involvement/engagement/participation” N/A AND “medical performance ”
Limits		
Dates	2004‐2018	
Language	English	
Location	International	
Article type	Academic	
Eligibility criteria		
Types of studies	All types of empirical studies (excluding reviews). Methodological quality—not used as an exclusion criterion but considered when synthesizing the evidence for all studies.	
Inclusion	**Population**: Regulation stakeholders; public, patients and doctors as well as the infrastructure for regulation; the national, regional (or federal) medical regulators or boards, professional bodies (eg, Royal colleges) and patient groups. **Intervention:** All forms of patient input: lay representation, PPGs, patient feedback, online reviews, information from patient surveys (experience/satisfaction), compliments and complaints. **Outcome**: Studies with an outcome linked to regulation or performance. **Study design:** All studies as above.	
Exclusion	1. Reviews/Commentaries/Non‐empirical etc. 2. PPI in clinical/treatment decision making/research/education (or training) 3. PPI in regulation/performance of other health professionals 4. PPI in the design of health service provision 5. Studies assessing the validity of patient feedback/satisfaction/experience tools	
Search interval	April 2016 – June 2016, updated in June 2018	

### 2.3 Search: Study selection

Electronic databases MEDLINE, PsycINFO and Google Scholar were systematically searched for articles published in the English Language between January 2004 and June 2018. Although this review considers the role of PPI in medical performance globally, we selected January 2004 as a start date, as around this time there was growing discussion of the role of PPI in future proposals for revalidation in the UK. Electronic database searches were supplemented with ancestry and forward citation searches. Two independent reviewers undertook the review process at each stage. Duplicate studies were removed electronically and double‐checked by a second researcher. Studies were selected using a two‐stage process. Firstly, all identified titles and abstracts were screened by each of the reviewers using previously agreed inclusion/exclusion criteria (Table 1). Articles of included abstracts were then reviewed independently by each reviewer in full and assessed against the eligibility criteria. Discrepancies were resolved by discussion or sent to a third reviewer until consensus was achieved.

### 2.4 Quality appraisal

An assessment of the quality of studies included in the review was undertaken to provide a comparative measure of study quality rather than for study exclusion, particularly as PPI as a singular intervention in medical performance processes is not consistently applied and given its relatively recent emergence, this review did not intend to evaluate its effectiveness. Nevertheless, to inform the robustness of the synthesis, quality assessment was undertaken using appropriate tools such as CASP for qualitative studies; an adapted version of a quality appraisal check list for case series studies; and the Newcastle‐Ottawa scale for observational studies (adapted for cross‐sectional studies).^26^, ^27^, ^28^

### 2.5 Data extraction, analysis and synthesis

Data extracted from eligible studies were organized by the first reviewer under the following headings: year of publication, country in which study was undertaken, population (eg, patients/doctors), intervention (eg, complaints), context, study design, summary of findings and key themes (see Table S1). The resulting table of included studies was verified by the second reviewer. An inductive approach was employed. A coding framework was developed and then used to individually analyse all included studies. The second reviewer independently coded a random sample of 25% to ensure coding accuracy. Identified themes were synthesized using a narrative approach following Popay et al guidelines. Popay et al describe narrative synthesis approach as “relying primarily on the use of words and text to summarize and explain findings from a synthesis.”

## 3 RESULTS

**Figure 1:**
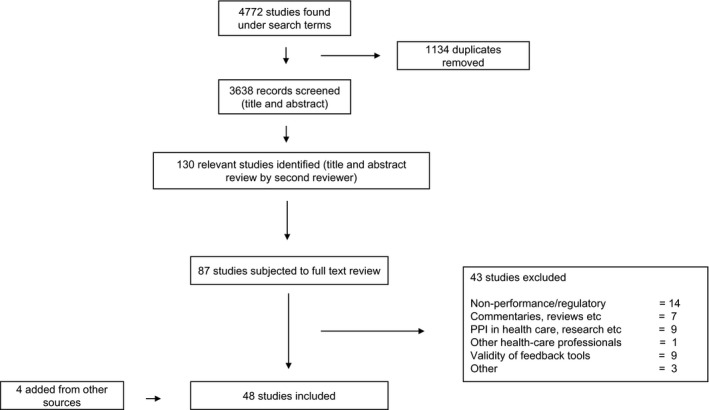
Summary of study selection process*—*4772 studies were initially identified using the search terms. Following PRISMA guidelines, the selection process resulted in a final 48 studies to be included in the review

The search identified 3638 articles (once duplicates had been removed). The titles and abstracts of these were screened and 87 were initially found to be relevant and full text versions were obtained. Following full text assessment and preliminary synthesis, 37 studies were excluded based on their outcome, not related to performance, leaving 48 studies that met the eligibility criteria (Figure 1). The key features of the included studies (categorized by study design, eg, cross‐sectional study) including publication title, year of publication, author, country in which the study was undertaken, type of PPI intervention (eg, complaints) and quality appraisal score are summarized in Table 2.

**Table 2:** Table summarizing the key characteristics of the included studies (categorized by type of study)

Title	Author	Year	Country	Intervention (type of PPI) for example complaints	Quality appraisal score
Case series studies (appraised using adapted version of Moga et al^27^ tool, scores given out of 13)					
Complaints, grievances, and claims against physicians: does tort reform make a difference?^67^	Alexander A	2010	United States	Complaints	9
Factors that might undermine the validity of patient and multi‐source feedback^45^	Archer J	2011	United Kingdom	Patient feedback	13
Patient complaint cases in primary health care: what are the characteristics of general practitioners involved?^68^	Birkeland S	2013	Denmark	Complaints	12
Characteristics of complaints resulting in disciplinary actions against Danish GPs^69^	Birkeland S	2013	Denmark	Complaints	10
Accountability sought by patients following adverse events from medical care: The New Zealand experience^40^	Bismark M	2006	New Zealand	Complaints	11
Relationship between complaints and quality of care in New Zealand: a descriptive analysis of complainants and non‐complainants following adverse events^35^	Bismark M	2006	New Zealand	Complaints	11
Identification of doctors at risk of recurrent complaints: a national study of healthcare complaints in Australia^42^	Bismark M	2013	Australia	Complaints	13
Formal complaints at an eye hospital: a three‐year analysis^55^	Chavan R	2007	United Kingdom	Complaints	7
Association of patient satisfaction with complaints and risk management among emergency physicians^52^	Cydulka R	2011	United States	Complaints/patient satisfaction	10
Evolution of patients’ complaints in a French university hospital: is there a contribution of a law regarding patients’ rights?^37^	Giugliani C	2009	France	Complaints	11
Colleague and patient appraisal of consultant psychiatrists and the effects of patient detention on appraisal scores^70^	Heneghan M	2016	United Kingdom	Patient feedback	7
Patient complaints and malpractice risk in a regional healthcare centre^53^	Hickson G	2007	United States	Complaints	9
Epidemiology of medical complaints in Mexico: identifying a general profile^71^	Jimenez‐Corona M	2006	Mexico	Complaints	11
One‐year audit of complaints made against a University Hospital Surgical Department^49^	Mann C	2012	United States	Complaints	6
Analysis of formal complaints in 1,645 liposuction operations^72^	Nathan B	2014	United Kingdom	Complaints	5
Increased number of ear‐nose‐throat malpractice complaints in Denmark^73^	Nikoghosyan‐Bossen G	2012	Denmark	Complaints	7
Relation of patients’ experiences with individual physicians to malpractice risk^74^	Rodriguez H	2008	United States	Patient experience/complaints	11
A 22‐month study of patient complaints at a National Health Service hospital^75^	Siyambalapitiya S	2007	United Kingdom	Complaints	6
The relation of patient satisfaction with complaints against physicians and malpractice lawsuits^46^	Stelfox H	2005	United States	Complaints/patient satisfaction	10
Patients’ complaints in a hospital emergency department in Singapore^76^	Wong L	2007	Singapore	Complaints	9
Cross‐sectional studies (appraised using adapted version of the Newcastle‐Ottawa assessment scale,^28^ out of 10)					
Patient feedback in revalidation: an exploratory study using the consultation satisfaction questionnaire^47^	Baker R	2011	United Kingdom	Patient feedback (experience)	4
The impact of complaints procedures on the welfare, health and clinical practise of 7926 doctors in the UK: a cross‐sectional survey^77^	Bourne T	2015	United Kingdom	Complaints	7
Factors associated with variability in the assessment of UK doctors’ professionalism: analysis of survey results^43^	Campbell J	2011	United Kingdom	Patient feedback	7
New Zealand doctors’ attitudes towards the complaints and disciplinary process^29^	Cunningham W	2004	New Zealand	Complaints	5
The immediate and long‐term impact on New Zealand doctors who receive patient complaints^78^	Cunningham W	2004	New Zealand	Complaints	5
Obtaining patient feedback at point of service using electronic kiosks^48^	Dirocco D	2011	United States	Patient feedback	5
Complaints handling in hospitals: an empirical study of discrepancies between patients’ expectations and their experiences^36^	Friele R	2008	Netherlands	Complaints	5
General practitioners’ experience and benefits from patient evaluations^56^	Heje H	2011	Denmark	Patient evaluations	7
Feedback on doctors’ performance from parents and carers of children: a national pilot study^34^	Mcgraw M	2012	United Kingdom	Patient feedback	6
Insightful practice: a reliable measure for medical revalidation^79^	Murphy D	2012	United Kingdom	Patient feedback	6
The response of doctors to a formal complaint^80^	Nash L	2006	Australia	Complaints	4
Obtaining patient feedback in an outpatient lithotripsy service is facilitated by use of a touch‐screen tablet (iPad) survey^51^	Turney B	2014	United Kingdom	Patient feedback	1
Qualitative (appraised using CASP qualitative^26^ studies checklist, scores out of 10)					
Challenges to the credibility of patient feedback in primary healthcare settings: a qualitative study^41^	Asprey A	2013	United Kingdom	Patient feedback	9
Patient involvement in a professional body: reflections and commentary^31^	Baker A	2007	United Kingdom	Lay involvement	1
Can GPs working in secure environments in England re‐license using the Royal College of General Practitioners revalidation proposals?^81^	Coomber J	2012	United Kingdom	Patient feedback	9
Defensive changes in medical practice and the complaints process: a qualitative study of New Zealand doctors^32^	Cunningham W	2004	New Zealand	Complaints	8
The medical complaints and disciplinary process in New Zealand: doctors’ suggestions for change^39^	Cunningham W	2004	New Zealand	Complaints	10
Experiencing patient‐experience surveys: a qualitative study of the accounts of GPs^33^	Edwards A	2011	United Kingdom	Patient feedback (experience)	10
Structuring patient and family involvement in medical error event disclosure and analysis^82^	Etchegaray J	2014	United States	Adverse events analysis	9
Motivators and barriers to using patient experience reports for performance improvement^30^	Geissler K	2013	United States	Patient experience	8
Multisource feedback questionnaires in appraisal and for revalidation: a qualitative study in UK general practice^44^	Hill J	2012	United Kingdom	Patient feedback	10
Content analysis of patient complaints^50^	Montini T	2008	United States	Complaints	7
Investigating complaints to improve practice and develop policy^83^	Parry J	2009	Australia	Complaints	8
Poor professionalism identified through investigation of unsolicited healthcare complaints^38^	Van Mook W	2012	Netherlands	Complaints	8
Patient complaints about physician behaviours: a qualitative study^57^	Wofford M	2004	United States	Complaints	7
Randomized control trials (appraised using CASP RCT checklist^26^, score out of 11)					
Real‐time patient experience surveys of hospitalized medical patients^84^	Indovina K	2016	United States	Patient feedback	8
Other (no tools available)					
Revalidation: Patients or process? Analysis using visual data^85^	Guillemin M	2014	United Kingdom	PPI overall	n/a
The use of patient complaints to drive quality improvement: an exploratory study in Taiwan^59^	Hsieh S	2010	Taiwan	Complaints	n/a

### 3.1 Quality appraisal

Quality appraisal of the included studies in this review was challenging for two reasons. Firstly, the heterogeneity of study designs used in the included studies limited comparison of study quality between studies. Secondly, the quality appraisal tools did not exist in an original format and either required adaptation or were not directly relevant for the studies they were designed to assess, for example CASP for qualitative studies when applied to content analysis of free text responses from surveys. Hence, we did not use quality appraisal results to draw any conclusions on the overall findings from this review. Quality appraisal scores are listed in Table 2.

A coding framework drawn from the data in the included studies was produced and primarily arranged into three overarching themes issues shaping PPI, mechanisms for PPI and impact of PPI on the systems and processes of medical regulation. Within these themes, emerging sub‐themes are presented with potential barriers and gateways for wider evolution or implementation of PPI models, based on the evidence for their positive and negative impacts, providing a narrative for PPI in different settings.

#### 3.1.1 Issues shaping patient and public involvement

The review has identified four main issues that shape PPI in medical performance processes relating to the individual doctor, the profession, the individual patient and the public; these are (a) the attitudes of the individual doctor (and profession), (b) patient characteristics, (c) the understanding of the purpose of PPI and (d) key relationships for PPI.

##### Attitudes of the doctor and profession

In some studies, the negative attitudes of doctors and the profession emerged as an important barrier, potentially hindering PPI from developing within systems and processes relating to medical performance.^29^, ^30^ For example, a study conducted by Baker et al^31^ which described lay involvement in a professional body in the UK concluded that the profession was guarded and favoured maintaining its boundaries with society, viewing patients as consumers of care, not as participants in the shared development of agendas. The article advocated for organizational structures to be modified to facilitate public accountability and to allow patients to become involved in agenda setting and decision making:

The new requirements for public accountability have been interpreted within a commercial syndrome, drawing on concepts of responsiveness to the individual patient as consumer. Wider issues of accountability, relating to the responsibility of the professional body in shaping the structures of health care, challenge the boundaries and rights of the profession defined within the guardian syndrome, and are much more difficult for a professional body to address.^31^

Conversely, positive attitudes were demonstrated to act as a gateway to PPI development. In two studies, doctors encouraged patient input into the complaints process whilst also suggesting that complaints data should inform the development of working practices so as to minimize future complaints.^29^, ^32^ Similarly, doctors were supportive of patient feedback citing it as important for developing relationships with patients, their families and even the local community.^33^

##### Patient characteristics

Patient characteristics may act as barriers, limiting patient access to feedback or complaints systems. For example, tools for patient feedback were deemed inappropriate for certain age groups, for example children,^34^ and access to and utilization of complaints systems were dependent upon age (older patients), socioeconomic status (low income) and ethnicity (minorities), with fewer complaints received from these groups, a specific concern raised from a study conducted in Australia and New Zealand.

The relatively low propensity to complain among patients who are elderly, socioeconomically deprived, or of Pacific ethnicity suggests troubling disparities in access to and utilisation of complaints processes. Further research is required to better understand and address these disparities.^35^

##### Perceptions of purpose of complaints and feedback

There appears to be divergence between patients and doctors, and among doctors as a group on the purpose of complaints and feedback. The differing conceptualization of this purpose is a potential barrier to developing PPI in medical performance processes. For example, one study cited ambiguity relating to the purpose of patient feedback; patients were unsure as to whether they were providing feedback on the service or the individual doctor.^33^ Some studies suggested that the purpose of complaints was to increase accountability and enhance professionalism in doctors.^36^, ^37^, ^38^ In contrast, some doctors and patients perceived complaints as a punitive measure that highlighted issues with performance or competency.^39^ In one study, patients suggested that disciplinary action against the doctor was not always the preferred outcome of lodging a complaint but, because complaints’ systems were perceived as inadequate and unable to provide the reassurance that was sought, patients felt that they had little choice but to pursue a litigious approach.

… behaviour reveals that injured patients seek manifold forms of accountability…This implies that systems that offer litigation as the key or sole mechanism for consumers to bring strong external oversight to bear on clinicians and hospitals may not respond to the wants of many patients. In such systems, a subset of plaintiffs will resort to litigation for lack of more fitting options.^40^

##### Key relationships for PPI

Two main relationships pertinent to PPI in medical regulation emerged: the doctor‐patient relationship and the profession‐public relationship. Our review found that complaints and negative feedback may compromise the doctor‐patient relationship but also provide an opportunity for improvement.^41^, ^42^, ^43^ One study discussed the potential for the development of a positive profession‐public/society relationship if the profession was willing to acknowledge the importance of society's right to complain.

The study indicates that doctors strongly support society's right to complain, having lay input into the process, achieving a sense of completion for both parties, and having those responsible for making decisions about complaints advised in an appropriate manner.^29^

#### 3.1.2 Mechanisms for patient and public involvement

Patient feedback was identified as a key mechanism for PPI in medical performance processes, especially in the UK. For doctors, the effectiveness of patient feedback tools was an important factor in the perceived value of the data obtained. This was associated with the validity of the tool and the reliability of the resulting data. Doctors in these studies suggested that patient feedback as part of MSF was a useful tool for formative improvement but queried the credibility of the data for performance or competency assessment.^33^, ^41^, ^44^ Concerns related to the internal validity of the tools including bias in selection of patients by a doctor (or members of staff) and in responses received from patients skewed towards providing more favourable feedback. Furthermore, the authors in these studies suggested that patient feedback scores did not always correlate with colleague feedback scores.

… concerns relating to aspects of methodology such as whether patients and colleagues can provide objective feedback may undermine its credibility as a tool for identifying poor performance.^44^

Although colleagues appear to report poor performance using MSF, patients fail to report concurrent findings. This challenges the validity of patient feedback as it is currently constructed.^45^

#### 3.1.3 Impact of patient and public involvement on the systems and processes of medical performance

The impact of PPI through complaints and feedback data (from patient experience, satisfaction and feedback surveys) can be viewed as both barriers and gateways to PPI development in medical performance processes, initiating both positive and negative changes to a doctor's practice.

##### Evaluating poor performance through a complaint or a negative patient experience

In a few of the included studies, authors concluded that complaints or negative feedback data could be used to evaluate poor performance.^30^, ^38^, ^46^ Baker et al proposed that patient feedback data provided an opportunity to identify doctors who needed educational support and possibly remediation.^47^

##### Quality improvement

A positive outcome of complaints and negative feedback data was the opportunity for quality improvement for both the individual doctor and the service through learning from previous issues, testing new ideas and implementing different approaches to limit future problems.^48^, ^49^, ^50^, ^51^ Many authors also perceived complaints as a conduit for managing “at risk” doctors, enabling organizations to mitigate risk through performance management.^52^, ^53^ Complaints and patient satisfaction data have been previously proposed as a useful quality improvement tool.^54^ Additionally, one study suggested that low patient satisfaction scores were a predictor for future complaints providing an opportunity to performance manage a doctor whilst enabling patients to participate in quality improvement.

There is wide consensus in the health care community on the need for regular monitoring and assessment of clinical performance and for public accountability. Physicians with dissatisfied patients represent an opportunity for quality improvement, and asking patients to evaluate physicians’ performance empowers patients to participate in quality improvement.^46^

Van Mook et al^38^ linked quality improvement to professionalism suggesting that complaints about perceived medical errors and complications were common, but the majority related to professional aspects of care, especially communication. Inadequate communication was frequently cited as a prominent reason for a complaint or negative feedback, along with a doctor's behaviour and approach to practice, all of which were aspects that could be improved to enable a better patient experience:

The concept of professionalism does encompass the entire continuum from the individual (attributes, capacities and behaviours), via the interpersonal (interactions of patients and healthcare professionals) to the macro‐social level (eg, institutional and social responsibility and economic imperatives).^38^

Both authors and study participants described complaints and patient feedback data as facilitators for learning and development.^55^, ^56^ Wofford et al^57^ suggested that learning from complaints as part of medical education may enhance professionalism in medical graduates. Additionally, identifying aspects of the doctor's interaction with patients (including their behaviour) or their practice that required improvement could be enabled through a process of reflection which may facilitate positive changes to practice.^44^

Conversely, complaints and patient feedback may have negative implications, acting as a barrier to PPI in developing medical performance processes, resulting in defensive practice with limited impact on delivery of care, to the detriment of the doctor‐patient relationship.

… findings that a complaint may adversely impact on the doctor's ability to practice medicine in a day‐to‐day setting is important…. There is no evidence from this study that the delivery of patient care is actually improved by the receipt of a complaint, and these results suggest that complaints against doctors have the potential to impact negatively upon patient care.^32^

## 4 DISCUSSION

This study has provided a systematic review and narrative synthesis of the international literatures on PPI in medical performance processes. The review has shown that PPI in medical performance processes is primarily through complaints and patient feedback with minimal patient input into the actual mechanisms. The review has produced a robust body of evidence identifying key gaps in the academic literature relating to PPI in medical performance processes in terms of (a) the extent of PPI, (b) shaping of the PPI agenda and (c) the impact of PPI on systems and processes.

In terms of shaping PPI in medical performance processes, a significant barrier identified was the doctor/profession attitude towards PPI. Whilst, for example, the General Medical Council in the UK established lay involvement at the uppermost levels of the organization well over a decade ago, PPI in regulatory processes is still largely through patient feedback. This review has shown that there is a need to establish the extent of PPI in medical performance processes. This is to ensure that the patient voice in the infrastructure and mechanisms of medical performance processes develop beyond lodging a complaint and completing a patient feedback or satisfaction form.

The focus for PPI has been described as being directed to regulatory strategy acting on the doctor/regulator relationship, rather than the doctor/patient relationship.^58^ However, this review has found a growing discourse about the role of patient input in the doctor/patient relationship. At this interface, complaints and feedback data are thought to initiate changes in practice by the individual doctor, both positive (quality improvement) and negative (defensive practice).^30^, ^32^, ^59^ Unintended and negative consequences such as defensive practice or the impact on a doctor's self‐confidence are potential risks to the quality of patient care.^29^, ^47^ Nonetheless, some within the profession acknowledge that patients have a role to play in complaints procedures.^29^ Addressing negative attitudes is challenging and reflects the current conceptualization of PPI in health care whereby some health professionals and organizations struggle to embrace the notion of partnership with patients and even feel threatened by the idea of active involvement, favouring consultation over collaboration.^60^

The review has shown that doctors view feedback and complaints as both a summative and formative assessment of their performance. In the included studies, doctors were particularly concerned about feedback and complaints data being used for summative assessment and in a minority of cases, doctors perceived complaints as a potentially punitive measure. If feedback and complaints were perceived as having a formative function, they may be viewed more favourably and the patient's view held in higher regard. In Alberta, Canada, patient feedback used for the purposes of recertification is mandated but data cannot be subpoenaed in a court of law and thus mitigates the perception that such data will be used for litigation purposes.^44^ Better advocacy of the purpose of complaints and feedback for doctors and patients may provide more meaningful insights for a doctor's practice. Contrary to conventional opinion, the findings from this review suggest that complaints are an integral part of PPI not just a reflection of wider systemic issues, although the challenge remains in disentangling their benefits (eg, quality improvement) from the common perception that they are solely critical feedback.

Where doctors viewed patient feedback and complaints data as having a developmental function, there were significant opportunities for quality improvement, improving performance and enabling professionalism. Organizationwide reporting and better coordination of complaints and feedback data that highlights performance issues may enable individual doctors and services more generally to improve the quality of care they provide. This may require a shift in culture that fosters organizational leadership and patient‐centred care creating an environment in which complaints and feedback form a key component of quality improvement initiatives as they are viewed positively by doctors, removing the fear of blame, so often perpetuated by negative feedback and complaints.^61^ Indeed, in other spheres of health care, PPI in quality improvement has been suggested as positively influencing organizational culture by increasing emphasis on non‐hierarchical, multidisciplinary collaboration, encouraging staff to model desired behaviours of recognition and respect, and commitment to rapid translation of research into practice.^62^

The barriers to PPI in medical performance processes identified in the review could also be viewed as opportunities. The existence of complaint systems in numerous countries is promising and provides a mechanism by which patients can participate in the assessment of a doctor's performance. Furthermore, the recognition of limited accessibility to feedback and complaints systems for certain demographic groups is also encouraging providing organizations and patient groups with “targets” for their advocacy. Older patients and those from certain ethnic backgrounds are less likely to lodge complaints or provide feedback on their doctor.^35^ Understanding the reasons for this is required to better engage these groups in PPI. Innovative approaches to patient feedback collection such as the use of touchscreens at the point of service may improve response rates as they are accessible and inclusive to most.^51^

The potential positive impacts of PPI outlined in this review such as promoting professionalism among doctors and improving the quality of care delivery require a greater focus in future research studies. Authors in some of the included studies focused on the reasons for complaints and feedback being less impactful, citing tools and data as limitations. This was exemplified by concerns of the credibility of patient feedback data with some doctors critiquing the design of tools, questioning the process of collecting data (selection bias) and the reliability of responses received from patients (response bias).^45^, ^63^, ^64^ This is despite tools having been repeatedly tested for their validity and generalizability, with reasonable evidence to suggest that they are reliable.^63^, ^65^

The review has identified the need for a better understanding of the actual impact of the different types of PPI in their current format in regulatory processes and systems, at the level at which patients participate in medical performance processes, that is through complaints and feedback both of which may indicate clinical, managerial and broader systemic issues or a deterioration in the doctor‐patient (or service‐patient) relationship.^66^ However, in this review complaints and negative feedback have been identified as possible conduits for individual doctor and service improvement. Thus, PPI has a potentially significant role in improving the quality, relevance and ultimately the value of complaint and feedback mechanisms, which is integral to promoting accountability and professionalism, thus enhancing the doctor‐patient relationship.

### 4.1 Strengths and limitations of this review

The uncertainty of the precise definition of medical performance somewhat hindered the assessment of studies for eligibility in this review; yet, the included studies focused on aspects closely associated with medical performance such as professionalism, competency and professional development. Even so, without a precise definition for medical performance it is possible some studies were missed. In an attempt to overcome this issue, the review encompassed the international literature on PPI in medical performance processes, including studies from several countries with different medical regulatory systems and approaches for assessing medical performance within which the extent of PPI was somewhat varied. Nonetheless, given the heterogeneity of contexts and systems it is challenging for this review to provide standardized recommendations for developing PPI in medical performance processes.

This study has used a robust approach to review the evidence for PPI in medical performance processes including a quality appraisal of included studies. Additionally, the use of a narrative synthesis is important as it has provided the opportunity to use words and text to summarize and explain findings from the reviewed literature thus providing evidence on the barriers and gateways to PPI in medical performance processes whilst highlighting the key evidence gaps that need to be addressed.

## 5 CONCLUSION

The significance and recognition of PPI have grown in many domains of health care in recent years propagating an evolution of “patient‐centred care” and shared clinical decision making. This review indicates a need for a similar level of integration for PPI within medical performance processes as existing models are both fragmented and inadequate to have a meaningful impact on systems and processes that assess and monitor performance.

Feedback and complaints have both summative and formative elements, though the balance varies between different systems and even within systems. PPI can make a positive contribution to developing both elements, although the evidence presented in this review suggests that most doctors would prefer patient feedback and complaints to provide a primarily formative assessment of their performance and are cautious about the use of such data for summative purposes. Developing the formative element of feedback and complaints mechanisms with patients involved in the design of their structures and systems may have a greater impact on the professional development of doctors.

More broadly, quality improvement may act as a driver for PPI in medical performance processes to evolve beyond the level of providing feedback and lodging complaints, forming the foundation of a transition from a culture of contractual PPI that exists as part of the clinical interface between the doctor and patient, to that of collaboration that enhances the profession‐society relationship.

## AUTHORS’ CONTRIBUTIONS

ML, RB and SRdB conceived and designed the review. ML, RB, SRdB, SM and SB developed, reviewed and approved search criteria. ML and RB undertook the review. ML wrote the first draft of the manuscript. ML, RB, SRdB, MB, JA and MM revised subsequent versions of the manuscript. All authors approved the final version.

## CONFLICT OF INTEREST

There are no known conflicts of interest.
